# Recurrent Periocular Nodules: Lymphomatoid Papulosis in an Uncommon Anatomic Site

**DOI:** 10.7759/cureus.88221

**Published:** 2025-07-18

**Authors:** Alexa Lum, Jessica Colon, Kristi Hawley

**Affiliations:** 1 Dermatology, Michigan State University College of Osteopathic Medicine, East Lansing, USA; 2 Internal Medicine, Memorial Healthcare System, Pembroke Pines, USA; 3 Dermatology, The Derm Institute of West Michigan, Caledonia, USA

**Keywords:** cd30-positive lymphoproliferative disorders, chronic lymphoproliferative diseases, cutaneous t-cell lymphoma (ctcl), lymphomatoid papulosis, papulonodular disease

## Abstract

Lymphomatoid papulosis (LyP) is a rare cutaneous lymphoproliferative disorder involving the dysregulation of CD30-positive T-cells that accumulate in the skin. The accumulation of CD30-positive T-cells leads to the development of papulonodular lesions, which typically follow a protracted course and often resolve spontaneously in a matter of days to weeks. This case report describes a 44-year-old male patient who initially presented to the dermatology office with recurrent nodules under the right eye. He was diagnosed with periocular dermatitis initially, but when his condition failed to improve with minocycline, he was subsequently diagnosed with papulopustular rosacea and impetigo. However, despite multiple treatment trials, he still experienced recurrent flares with nodules that would arise only under the right eye and subsequent pain, swelling, and inflammation, which subsided as the lesions resolved. Histopathology of one of the lesions revealed a CD30-positive atypical lymphoid infiltrate, consistent with a diagnosis of LyP. Due to its close resemblance to other lymphoproliferative diseases, a diagnosis of LyP requires characteristic histological findings and a compatible clinical presentation, as neither alone is sufficient for diagnosis. This case report highlights the importance of integrating both clinical presentation and histopathologic findings to establish a diagnosis of LyP, especially when it arises in an uncommon anatomic site. LyP typically follows a benign course, but all patients diagnosed with LyP should have regular visits with a hematologist/oncologist to monitor for potential progression to malignant lymphoma.

## Introduction

Lymphomatoid papulosis (LyP) is classified as a rare cutaneous lymphoproliferative disorder involving dysregulation of CD30-positive T-cells that accumulate in the skin. The estimated incidence of LyP is approximately 1.2-1.9 cases per 1,000,000 people [1]. The peak incidence is typically in the fifth decade, with men being more affected than women [2].

The accumulation of CD30-positive T-cells causes an eruption of papulonodular lesions to form, which may eventually become hemorrhagic and erode [2]. These lesions typically present as either a single lesion or in multiple crops and can occur on any part of the body, most often on the trunk and extremities. They typically tend to wax and wane over a period of two to six weeks, often resolving spontaneously and forming atrophic scars [3]. Additionally, patients with LyP do not commonly experience systemic symptoms and do not display any lymphadenopathy.

There are two main variants of LyP that can be classified histologically: LyP type A and LyP type B. LyP type A, the more common variant, is characterized by a wedge-shaped dermal infiltrate composed of neutrophils, eosinophils, histiocytes, and large CD30-positive atypical cells resembling Reed-Sternberg cells [4]. In contrast, LyP type B is characterized by CD30-negative atypical cerebriform mononuclear cells, similar to those observed in mycosis fungoides [4]. A less common variant, LyP type C, resembles LyP type A in its large clusters of CD30-positive cells but differs by its markedly reduced inflammatory cell infiltrate [5].

Macaulay coined the term LyP in 1968 to describe the dermatologic condition characterized by papulonodular and papulonecrotic lesions that spontaneously resolve, exhibit histologic features resembling malignant lymphoma, and follow a benign, yet chronic, clinical course [6]. Despite the mainly benign clinical course of LyP, patients exhibit a higher risk of developing malignant lymphoma. Due to the higher risk of malignancy in patients with LyP, it is essential for clinicians to refer these patients to hematology and oncology for regular evaluation and management.

Treatment of LyP is guided by the number, size, and overall distribution of lesions. Typically, observation is sufficient to monitor the disease. Standard treatment regimens include topical steroids, photochemotherapy with psoralen ultraviolet A (UVA) light therapy, and low-dose methotrexate [7]. Other less commonly used treatments that have demonstrated benefit in LyP patients include topical tacrolimus, isotretinoin, photodynamic therapy, topical or systemic bexarotene, imiquimod 5% cream, and UVA/ultraviolet B therapy [7].

## Case presentation

**Figure 1:**
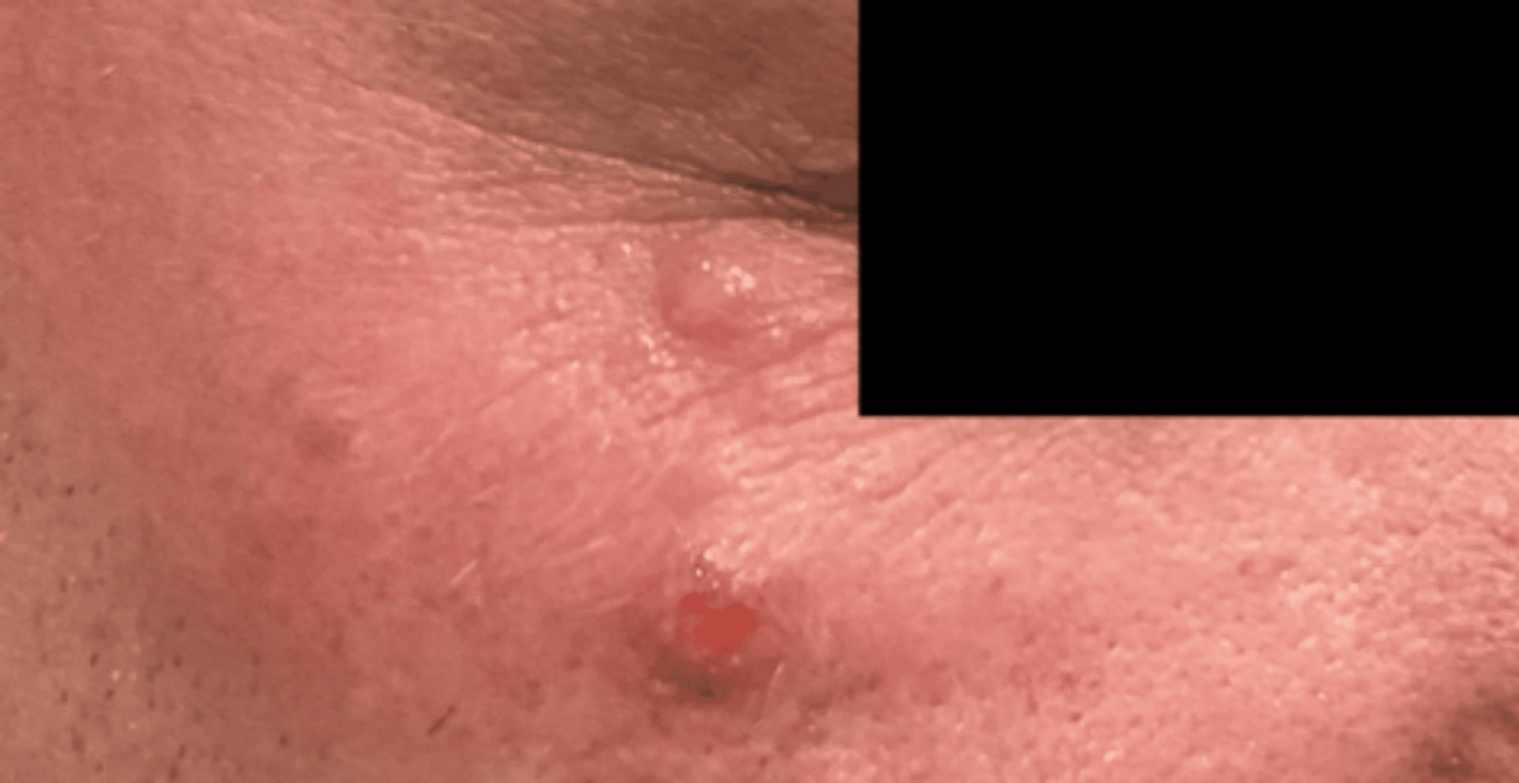
Painful and nonspecific erythematous nodules under the right eye

**Figure 2:**
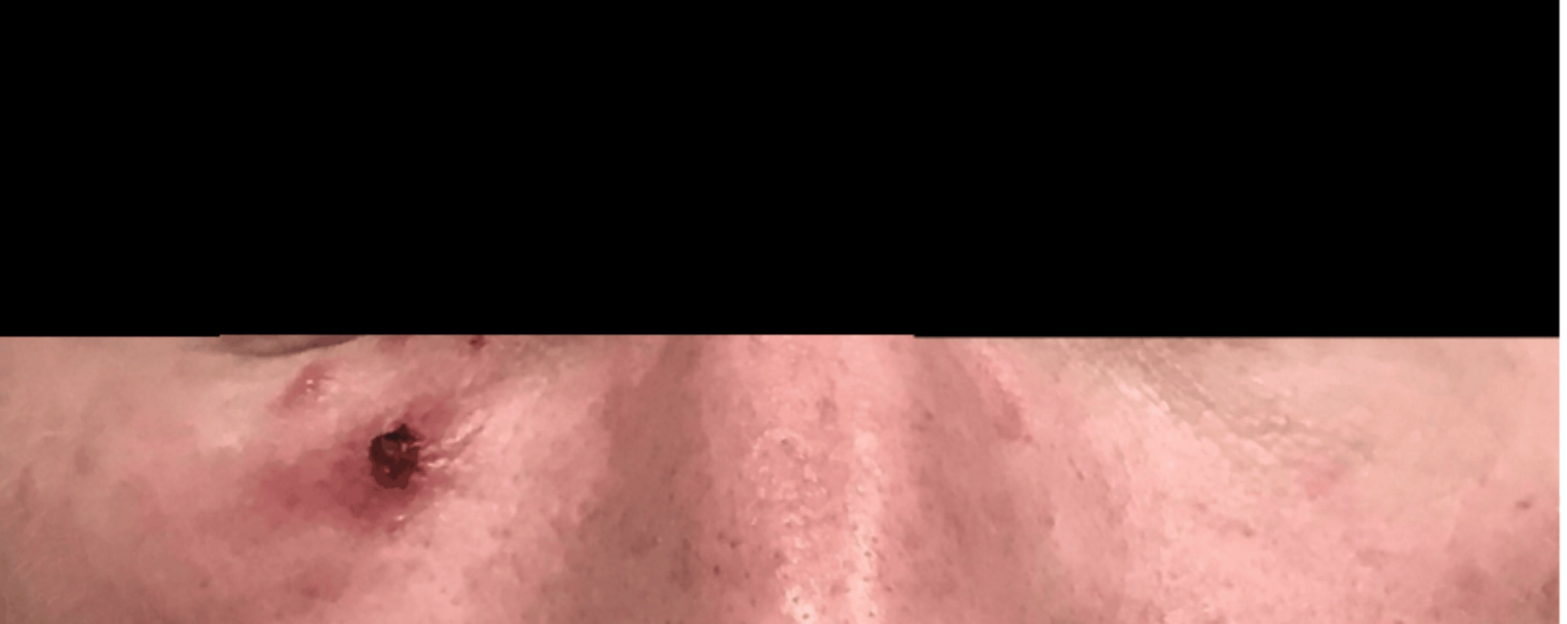
An erythematous nodule of 1 cm with a hemorrhagic center under the right eye

A 44-year-old male patient initially presented to the dermatology clinic with recurrent nodules under the right eye, reporting a similar episode the prior year that resolved spontaneously. He was subsequently diagnosed with periocular dermatitis and initiated on minocycline 100 mg daily. After three months, he reported mild improvement but continued to develop intermittent papules and nodules. Therapy was transitioned to topical metronidazole 0.75% gel to treat possible papulopustular rosacea. One year later, he experienced worsening lesions characterized by painful, inflamed, open sores under the right eye (Figure 1). These changes were accompanied by swelling, but no visual disturbances were observed. The treatment regimen was modified to a short course of systemic steroids, doxycycline 100 mg twice daily, mupirocin ointment applied twice daily, and intranasal fluticasone. After two weeks, symptoms improved moderately, but he reported that his nocturnal eye rubbing resulted in lesion rupture, pain, and inflammation, limited to the right side. Over subsequent months, recurrent nonspecific nodules continued to wax and wane under the right eye, despite multiple treatment trials. At the patient’s most recent visit, he exhibited a 1 cm red nodule with a hemorrhagic center under his right eye (Figure 2). The patient denied any foreign travel or any significant health issues. Ultimately, after nearly two years of recurrent flares, a punch biopsy and viral swab were performed. The histopathological results revealed a dense perifollicular mixed inflammatory infiltrate with eosinophils and a CD30-positive atypical lymphoid infiltrate, with no evidence of viral cytopathic changes or increased Demodex mites. Clinical and histopathological findings were most consistent with LyP. Unfortunately, histopathological slides depicting this were not obtained due to laboratory disposal. The patient was subsequently initiated on clobetasol 0.05% cream for symptom control and was ultimately referred to oncology for further evaluation and management.

## Discussion

Diagnostic challenges and mimickers

The diagnostic challenges posed by LyP are well recognized, particularly given its clinical overlap with more common inflammatory dermatoses, such as periocular dermatitis and papulopustular rosacea. The differential includes, but is not limited to, arthropod bites, atopic dermatitis, scabies, herpes simplex infection, molluscum contagiosum, lymphomatoid drug eruption, and syphilis [5]. These mimickers often lead to misdiagnosis and delayed treatment, as exemplified in this case, where the patient’s persistent periocular nodules were initially attributed to inflammatory etiologies and managed with multiple topical and systemic therapies with no significant improvement. To mitigate the risk of misdiagnosis, clinicians should maintain a high level of suspicion for LyP and monitor for characteristic features, such as recurrent, self-resolving papulonodular lesions.

Furthermore, LyP has the ability to mimic multiple malignant dermatological conditions such as pityriasis lichenoides et varioliformis acuta (PLEVA), transformed mycosis fungoides, and Hodgkin lymphoma [5]. The tendency for lesions to spontaneously regress is a distinguishing feature of LyP, as other lymphoproliferative diseases that produce tumors with CD30-positive expression, such as primary cutaneous anaplastic large cell lymphoma, typically produce larger and nonregressing lesions [2]. PLEVA, like LyP, can also present with papules that ulcerate. However, there tends to be absent or minimal expression of CD30 antigen in PLEVA lesions, whereas a more robust expression of CD30 antigen is seen in LyP lesions [8]. Additionally, it is crucial to differentiate LyP from other cutaneous T-cell lymphomas, such as mycosis fungoides. Unlike LyP, a CD30-positive lymphoproliferative disease that typically follows a benign course and is associated with the potential development of malignant lymphoma, mycosis fungoides is a CD4-positive T-cell lymphoproliferative disease that is considered a primary lymphoma and can involve the lymph nodes and internal organs [9].

Anatomic rarity and location analysis

Such diagnostic uncertainty is further compounded by the variable clinical presentation of LyP, which can range from a few scattered papules to widespread, recurrent nodules. Anatomically, LyP most frequently develops on the trunk and extremities, with acral and facial involvement, especially periocular, being exceedingly rare and not reported as commonly in the literature. LyP rarely presents in a segmental or localized manner and more commonly manifests in a generalized distribution [10]. In the case of our patient, the disease presented atypically with nodular lesions developing in a segmental pattern solely under the right eye. The periocular localization seen in this patient is, therefore, highly unusual and expands the known spectrum of LyP presentation, underscoring the importance of considering rare cutaneous lymphoproliferative disorders even in atypical sites.

Critical clinical implications

Importantly, this case highlights the critical need for early biopsy in patients with chronic, relapsing, or atypical lesions, particularly those unresponsive to standard therapies. Timely recognition of LyP is crucial due to its association with an increased risk of development of malignant lymphomas, such as CD30-positive anaplastic large cell lymphoma, Hodgkin lymphoma, and mycosis fungoides [10]. In a study investigating the development of associated lymphomas in patients with LyP, 34.2% of patients developed lymphoma before their diagnosis of LyP, 24.6% concurrently, and 41.2% following the diagnosis [2]. Additionally, histopathological evaluation is essential for distinguishing LyP from its mimics and for identifying the characteristic CD30-positive atypical lymphoid infiltrate, which is necessary for accurate diagnosis and appropriate management.

## Conclusions

This case underscores the diagnostic complexity and clinical significance of LyP, particularly when it presents in rare anatomical locations, such as the periocular region. The patient’s prolonged course and resistance to standard therapies underscore the importance of considering LyP in the differential diagnosis of persistent, recurrent cutaneous lesions, especially when they do not respond as expected. Accurate diagnosis relies on a combination of clinical suspicion and histopathological confirmation of CD30-positive atypical lymphoid infiltrates. Given the risk of progression to malignant lymphoma, regular follow-up and multidisciplinary management are essential. Ultimately, this case broadens awareness of the varied presentations of LyP and reinforces the need for early biopsy and coordinated care to optimize patient outcomes.
